# Analysis of the Effect of Demographic Variables on Lysosomal Enzyme Activities in the Missouri Newborn Screening Program

**DOI:** 10.3390/ijns11020048

**Published:** 2025-06-19

**Authors:** Lacey Vermette, Jon Washburn, Tracy Klug

**Affiliations:** 1 Missouri State Public Health Laboratory, P.O. Box 570, Jefferson City, MO 65102, USAtracy.klug@health.mo.gov (T.K.); 2 LaCAR US, 25 Alexandria Way, Durham, NC 27713, USA

**Keywords:** lysosomal storage disorders, demographic variables, age at sample collection, gestational age

## Abstract

Newborn screening laboratories are increasingly adding lysosomal storage disorders (LSDs), such as Mucopolysaccharidosis I (MPS I) and Pompe disease, to their screening panels. Without newborn screening, LSDs are frequently diagnosed only after the onset of symptoms; late detection can lead to profound and irreversible organ damage and mortality. While screening of these disorders has accelerated over the past five years, there is little published information regarding the potential correlation of demographic variables (age at sample collection, birthweight, gestational age, gender, etc.) with lysosomal enzyme activity. The Missouri State Public Health Laboratory prospectively screened more than 475,000 newborns for MPS I, Pompe disease, Gaucher disease, and Fabry disease between 15 January 2013 and 15 May 2018. This report investigates trends between several demographic variables and activities of four lysosomal enzymes: α-L-iduronidase (IDUA), acid α-glucosidase (GAA), acid β-glucocerebrosidase (GBA), and acid α-galactosidase (GLA). This information provides a valuable resource to newborn screening laboratories for the implementation of screening for lysosomal storage disorders and the establishment of screening cutoffs.

## 1. Introduction

Many newborn screening (NBS) programs in the U.S. and worldwide have recently implemented screening for lysosomal storage disorders (LSDs). LSDs are a group of approximately 50 rare, inherited metabolic disorders that are typically caused by single-enzyme deficiencies in the lysosomes. While individual LSDs are rare, the combined estimated incidence for any LSD is between 8 and 25 per 100,000 [1]. Two LSDs, Pompe and Mucopolysaccharidosis Type I (MPS I), were added to the U.S. Recommended Uniform Screening Panel (RUSP) in 2015 and 2016, respectively. While only two LSDs are currently included on the RUSP, several states have implemented screening of additional LSDs. Forty-three states and the District of Columbia are currently screening for at least one LSD [2], and several other states are expected to begin population screening soon. NBS assays for LSDs measure enzyme activity in newborn dried blood spot (DBS) specimens to identify enzyme deficiencies that may be indicative of an LSD.

Without newborn screening, LSDs are typically diagnosed through a combination of diagnostic enzyme assays, measurement of biomarkers in blood or urine, and molecular analysis; however, this generally occurs only after presentation of symptoms of disease progression. Diagnosis of LSDs can be challenging as the symptoms are often subtle and non-specific; average time from symptom presentation to diagnosis can range from 1.4 months (in the case of infantile-onset Pompe disease) [3] to over 10 years (in the case of adult Fabry disease) [4]. Early intervention prior to the presentation of symptoms can prevent or delay irreversible organ damage and mortality. Significant improvements in available therapeutic strategies have recently been made that have improved the quality of life for those with LSDs. Treatment strategies for LSDs vary by disease, but can include enzyme replacement therapy (ERT), small-molecule therapy, gene therapy, and/or hematopoietic stem cell transplantation (HSCT) [5].

The Missouri State Public Health Laboratory (MSPHL) initiated universal newborn screening for MPS I, Pompe, Gaucher, and Fabry disorders on 15 January 2013, in response to a legislative mandate to expand newborn screening to include LSDs. MSPHL was the first U.S. state laboratory to begin population-based screening for any of these disorders. With little published information available regarding lysosomal enzyme activity trends in newborns, the MSPHL performed careful analyses of trends between lysosomal enzyme activity and demographic variables. These analyses prompted changes in the screening workflow and screening cutoffs in Missouri. This manuscript summarizes the findings through five years of prospective newborn screening, including analyses of trends between lysosomal enzyme activities and birthweight, gestational age, gender, and age at sample collection.

## 2. Materials and Methods

### 2.1. Screening Procedure

All specimens were collected through the statewide newborn screening program. The MSPHL utilizes digital microfluidic fluorimetry (DMF-F) (SEEKER; Baebies, Inc., Durham, NC, USA) for screening of MPS I, Pompe, Gaucher, and Fabry diseases. The test quantifies activity of the α-L-iduronidase (IDUA), acid α-glucosidase (GAA), acid β-glucocerebrosidase (GBA), and acid α-galactosidase (GLA) enzymes in newborn dried blood spots. Decreased activity of these enzymes indicates an increased risk of MPS I, Pompe, Gaucher, and Fabry disorders. As the MSPHL was the first to begin prospective screening for these four disorders, the MSPHL initially established cutoffs based on 29 diagnostic samples from patients with one of the four disorders. These included newborn and non-newborn specimens, as well as specimens from samples with different forms of the disorders (both infantile- and late-onset Pompe, for example). Additionally, a pilot study was completed, which tested more than 13,000 samples, including premature newborns [6].

### 2.2. Establishment of Cutoffs

The MSPHL generally used three cutoffs for each enzyme: an instrument cutoff (highest), a borderline cutoff, and a referral cutoff (lowest). When each sample was received by the lab, the initial screening result was evaluated relative to the instrument cutoff. If the initial result was below the instrument cutoff, the sample was repeated in duplicate; if the initial result was above the instrument cutoff, the sample was presumed normal, and no further testing was required. For samples that were repeated in duplicate, the average activity of the three test results was evaluated relative to the referral and borderline cutoffs. If the average activity was above the borderline cutoff, the specimen was presumed normal. If the average activity was between the referral cutoff and the borderline cutoff, the lab requested an additional sample from the newborn. If the average activity was below the referral cutoff, a risk assessment was performed including evaluation of the other lysosomal enzymes. Specimens with multiple lysosomal enzymes below the screening thresholds may be indicative of poor sample quality; such specimens were classified as low risk, and an additional specimen was requested. Specimens that were not considered low risk were sent to the regional confirmatory referral center for follow up. The position of the cutoffs relative to the distribution has been described in a prior publication [7].

As data were collected through prospective screening, the initial cutoffs were modified after approximately six months to better reflect the distribution of samples received by the lab. Based on data indicating that median enzyme activity decreased throughout the first 30 days after birth for the GAA, GBA, and GLA enzymes, the MSPHL implemented discrete cutoffs for samples collected less than 7 days after birth, 7–13 days after birth, or more than 14 days after birth. Additionally, the MSPHL determined that the lysosomal enzyme activities exhibited seasonal variability; the median enzyme activities measured during the summer months were lower than during the winter months, which led to higher rates of false positive results during the summer. As a result, the MSPHL began periodically adjusting the cutoffs based on seasonal variability to improve assay specificity.

### 2.3. Analysis Procedures

The screening results of all specimens tested between 15 January 2013 and 15 May 2018 were included in this analysis; however, samples designated as poor quality by the laboratory upon receipt and accessioning were excluded from the analysis [8]. A total of 477,870 samples were included in the raw dataset. For samples that were tested multiple times, the mean of the individual test results was calculated and used in the analyses. The quantitative LSD enzyme screening results were merged with demographic information, which was recorded on the sample card at the time of dried blood spot sample collection and entered into the laboratory information management system (LIMS) at the time of sample receipt. The available demographic information included the age of the newborn at sample collection (in hours), gestational age (in weeks), birthweight (in grams), and gender.

Preliminary calculations and filtering were completed in Microsoft Excel; analysis and calculation of confidence intervals were completed in Minitab version 22.0 statistical analysis software (State College, PA, USA).

When analyzing each demographic variable (birthweight, gestational age, gender, and age at collection), efforts were taken to isolate other variables to avoid confounding the analyses. For example, the analyses for gender, gestational age, and birthweight were restricted to samples collected between 24 and 71 h after birth. The following sections detail exclusions from each dataset.

#### 2.3.1. Gender

From the starting dataset of 477,870 specimens, analysis of enzyme activity trends with gender was restricted to newborns born between 38 and 42 weeks of gestational age. Samples collected following blood transfusion were excluded from the analysis, as were specimens where the gender was not recorded. After exclusions, the dataset contained 302,328 specimens.

#### 2.3.2. Gestational Age

Samples in this cohort were excluded when gestational age was not recorded on the collection form. Additionally, samples where the recorded gestational age was less than 22 weeks or greater than 43 weeks were excluded, due to very small counts of specimens in those groups. After exclusions, the dataset contained 378,380 specimens. The groups at each extreme (22, 23, and 43 weeks) had the fewest number of samples (30, 147, and 36 samples, respectively). All other subgroups contained at least 294 samples. The 38, 39, and 40-week subgroups each had more than 60,000 samples.

#### 2.3.3. Birthweight

Birthweight, which was recorded in grams, was rounded down to the nearest 250 g for analysis, and samples were excluded when birthweight was not recorded. Additionally, samples where the recorded birthweight was less than 250 g or greater than 5750 g were excluded. After exclusions, the dataset contained 375,910 samples. The groups at each extreme (250, 5250, and 5500 g) had the fewest number of samples (98, 67, and 30 samples, respectively). All other subgroups contained at least 318 samples.

#### 2.3.4. Age at Collection

In Missouri, age at sample collection is recorded on the sample collection form in terms of hours. For this analysis, the age in hours was rounded down to the nearest full day. For example, a sample collected at 47 h would be rounded to one day. This analysis was limited to specimens collected from full-term births (38–42 weeks of gestational age) and from newborns who had not received blood transfusion. Samples collected at more than 30 days after birth were also excluded from the analysis. After exclusions, the dataset contained 337,886 samples. All subgroups contained at least 225 samples; subgroups between 0 and 17 days each contained at least 1019 samples.

## 3. Results

### 3.1. Distribution Shape

When analyzing each dataset, it was observed that the distributions for each lysosomal enzyme were generally lognormal for the entire dataset as well as for each subpopulation (for example, for the entire GAA population as well as specifically for samples collected between 38 and 42 weeks of gestational age). Because the distributions were non-normal, descriptive statistics such as mean and standard deviation could not be used to characterize the data—and typical methods to compare the means of different groups that are dependent on calculated variance within the subpopulations could not be completed without first transforming the data.

**Figure 1:**
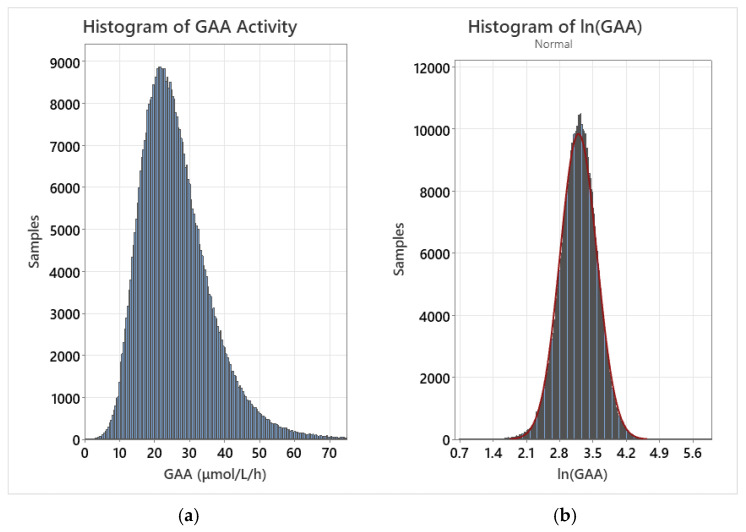
(**a**) GAA activity for the entire dataset; (**b**) GAA activity for the log-normalized dataset with overlaid normal fit (red line).

A log transformation was performed for each dataset by calculating the natural logarithm of all individual values. Following the transformation, the population distribution was closely approximated by a normal fit. As confounding variables were removed, the normal fit improved for the log-normalized distribution. For example, the transformed normal distribution fit improved when early preterm births and post-term births were removed from the dataset. Histograms of the raw GAA data for the entire dataset as well as the log-normalized dataset are displayed in Figure 1.

### 3.2. Calculation of Confidence Intervals

After transformation, descriptive statistics were calculated for each of the datasets. A confidence interval was calculated for each subgroup using individual (non-pooled) variances. The mean and 95% confidence interval for each subgroup were then back-transformed by calculating the natural exponent of each value. This allowed for the back-transformed means and confidence intervals to be represented on the normal activity scale. As the confidence intervals were calculated using normalized data, they are asymmetrical.

### 3.3. Gender

**Figure 2:**
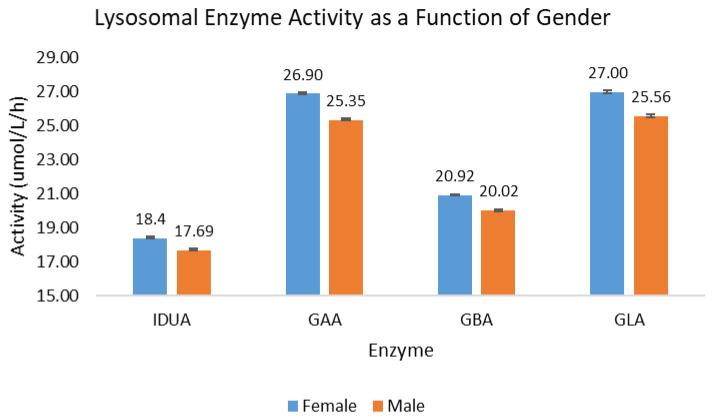
Lysosomal enzyme activity as a function of gender. Blue bars represent female births; orange bars represent male births.

Of the 302,328 samples included in the analysis of activity as a function of gender, a slightly larger percentage of the samples were identified as males (50.8%) as opposed to females (49.2%). For all four lysosomal enzymes, the back-transformed mean activity for females was between 3.9% (IDUA) and 5.8% higher (GAA) than for males. The back-transformed mean activity is presented in Figure 2; the error bars indicate the 95% confidence interval of the mean.

### 3.4. Gestational Age

**Figure 3:**
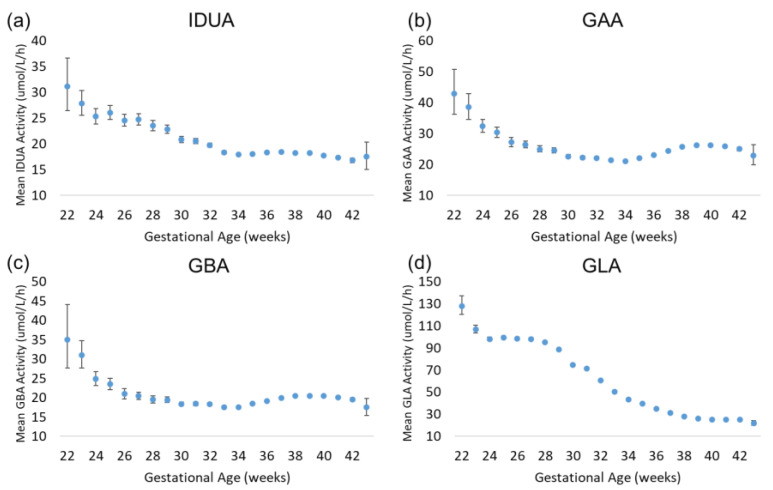
Back-transformed mean enzyme activity as function of gestational age: (**a**) IDUA, (**b**) GAA, (**c**) GBA, and (**d**) GLA. Error bars represent 95% confidence interval for each group. Data are distributed by week of gestational age (groups of 22–43 weeks).

Approximately 89.5% of the specimens included in the analysis were collected between 37.0 and 41.9 weeks of gestational age. These specimens represent early-term (37.0–38.9 weeks), full-term (39.0–40.9 weeks), and late-term (41.0–41.9 weeks) births. The rate of preterm births among our dataset is consistent with the 2007–2014 Centers for Disease Control and Prevention (CDC) preterm birth (<38 weeks) estimate of 9.54% [9]. The results of the enzyme analysis are displayed in Figure 3. For all four assays, there was a trend of increased enzyme activity in extremely premature births relative to early-term, full-term, and late-term births. For IDUA, GAA, and GBA, the increase was modest relative to full term (30–50% greater than full term); for GLA, the increase was 300% relative to full term. IDUA, GAA, and GBA mean activity decreased with increasing gestational age to approximately 34 weeks. At 34 weeks, mean IDUA activity continued to decrease slightly through 42 weeks, while mean GAA and GBA activity increased slightly until reaching full term. For GLA, the mean activity remained elevated through 28 weeks of gestational age, then gradually decreased through full term.

### 3.5. Birthweight

In this dataset, 8.13% of specimens were collected from newborns considered to be of low birthweight (LBW; less than 2500 g)—and 1.28% were collected from newborns considered to be of very low birthweight (VLBW; less than 1500 g). These birthweight trends are consistent with the 2017–2018 national averages reported by the CDC (8.28% and 1.38% for LBW and VLBW, respectively) [10].

**Figure 4:**
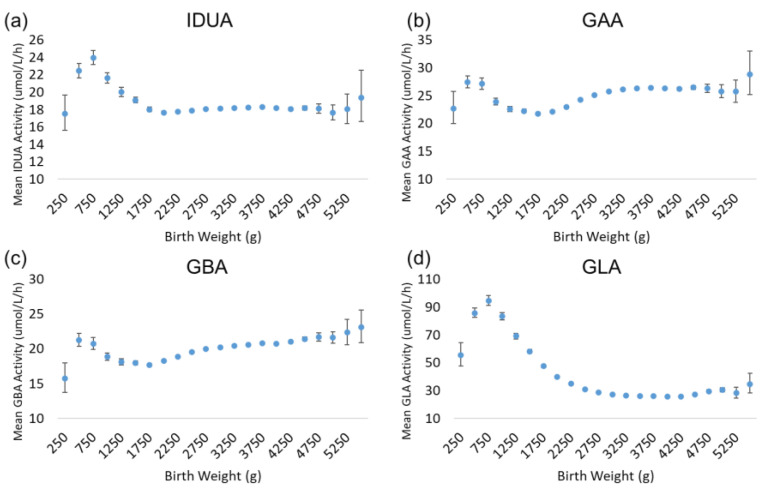
Back-transformed mean enzyme activity as function of birthweight: (**a**) IDUA, (**b**) GAA, (**c**) GBA, and (**d**) GLA. Error bars represent 95% confidence interval for each group. Data are distributed in groups of 250 g of birthweight (groups of 250–5500 g).

There is a very strong and well-characterized relationship between birthweight and gestational age [11,12,13,14]; generally, birthweight increases non-linearly with increasing gestational age. Since these variables are very closely related, the trends for enzyme activity as a function of birthweight are very similar to those observed for gestational age. Figure 4 illustrates the results for the analysis by birthweight. While the highest activity values for each enzyme were apparent in the lowest gestational age group (22 weeks), the back-transformed mean activities for the lowest birthweight group (250–499 g) were low relative to the subsequent groups. For all enzymes, activity increased transiently in newborns in the 500–749 g weight group. IDUA, GAA, and GBA activity declined in the 750–999 and 1000–1249 g weight groups, before reaching a baseline that remained stable in the larger weight groups. GLA activity exhibited a more gradual decline across the 1000–1249 to 2250–2499 g weight groups, before reaching a baseline low level of activity.

### 3.6. Age at Collection

Missouri’s state newborn screening program requires that a single dried blood spot specimen should be collected from each newborn between 24 and 72 h after birth, with a strong preference for samples collected between 24 and 48 h after birth. Consequently, 89.6% of the specimens in this dataset were collected between either 24 and 47 (82.0%) or 48 and 71 h (7.6%) after birth. While repeat specimens are often required in the case of premature births, this dataset was limited to specimens collected from newborns that were 38–42 weeks of gestational age at birth and thus excluded such repeat specimens. While this approach excluded a significant number of samples in the interest of isolating age at sample collection, the number of samples in the study prior to exclusion was so large (477,869 samples) that at least 225 samples were included in the analysis per day of collection. This allowed for tight confidence intervals for the back-transformed mean throughout the entire 30 days.

**Figure 5:**
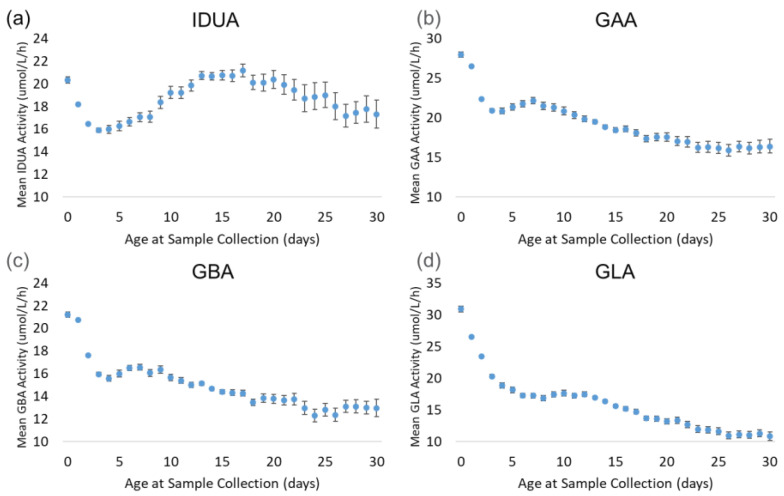
Back-transformed mean enzyme activity as function of age at sample collection (days): (**a**) IDUA, (**b**) GAA, (**c**) GBA, and (**d**) GLA. Error bars represent 95% confidence interval for each group. Data are distributed by day (groups of 0–30 days).

Figure 5 displays the trends of each enzyme by age at sample collection. The back-transformed means for all four enzymes decreased sharply from day 0 (hours 0–23) through day 3 (hours 72–95). During this period, the mean decreased by 22% (IDUA) to 34% (GLA). IDUA mean activity increased from day 2 through day 17, reaching the same activity as on day 0, before gradually decreasing through the end of the month. For GAA and GBA, the enzyme activity recovered slightly from day 3 through day 7, then decreased consistently through day 30. At day 30, the mean activity for GAA and GBA was approximately 40% lower than at day 0. The GLA mean decreased consistently from day 4 through day 30; by day 30, the mean activity decreased by 65% relative to the day 0 mean.

## 4. Discussion

Analyses of lysosomal enzyme activity by birthweight, gestational age, gender, and age at sample collection have been previously reported [15,16]; however, the analysis techniques differed somewhat from those used in this study. Additionally, this dataset included significantly more specimens than any other published studies. The Illinois Department of Public Health (IDPH) previously presented analyses of birthweight and age at sample collection for five lysosomal enzymes, while the Belgium Detection Center for Metabolic Diseases (PCMA) reported analyses of gestational age on IDUA and GAA enzyme activities. While many trends in those reports were confirmed by this study, several additional trends were also observed.

### 4.1. Gender

While the back-transformed means for females are approximately 5% higher than for males, the distribution shapes for both subpopulations are nearly identical. When evaluating the 1%, 5%, 10%, and 25% percentile for each assay and subpopulation, the activity for the male subpopulation at each percentile was lower than for the female subpopulation by 3.7% to 6.8%. These data suggest that newborn males generally have ~4–6% lower lysosomal enzyme activity than females across the distribution of each population.

### 4.2. Age at Collection

While Missouri only requires a single newborn dried blood spot sample to be collected between 24 and 72 h after birth from healthy full-term newborns, almost 10% of the samples received by the lab were collected outside of that time window. Therefore, it is critical to understand the potential relationship that age at collection has on enzyme activities—and to implement age-specific cutoffs when appropriate. Three of the enzymes—GAA, GBA, and GLA—exhibited pronounced decreases in mean enzyme activity through the first month after birth. If samples received throughout the month are evaluated using the same cutoff value, the screen positive rate for specimens received later in the month would be much higher. For example, the first percentile of the GLA distribution for samples collected one day after birth is 10.07 µmol/L/h; this indicates that 1% of specimens collected at age 1 day will have activity below 10.07 µmol/L/h. However, 9.8% of specimens collected on day 14 and 38.3% of specimens collected on day 28 had GLA activity below 10.07 µmol/L/h. Based on these reference ranges, a provisional cutoff of 10.07 µmol/L/h for GLA would result in a much larger percentage of later collection age samples falling below the cutoff. This example illustrates the importance of considering age-related cutoffs for GAA, GBA, and GLA, especially for states that utilize two temporally distinct dried blood spot specimens from each newborn.

**Figure 6:**
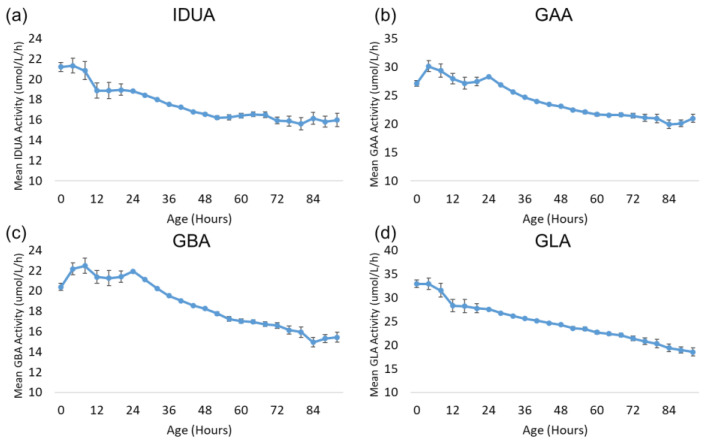
Back-transformed mean enzyme activity as function of age at sample collection (hours): (**a**) IDUA, (**b**) GAA, (**c**) GBA, and (**d**) GLA. Datapoints represent mean activity over four-hour block of time. Error bars represent 95% confidence interval for each subgroup. Data are distributed in groups of age in four-hour intervals (groups from 0 to 96 h).

Also of interest is the change in lysosomal enzyme activity during the first 96 h after birth. Since most specimens were collected during this time window and since all four enzymes exhibited a profound decrease in activity during this time frame, this period was examined with more granularity. Figure 6 displays the trends for each enzyme over the first 96 h after birth. The age at collection for each sample was rounded down to the nearest multiple of four hours to increase the number of specimens in each group and reduce the confidence intervals. All other aspects of the analysis were identical to the analysis by day of collection described above. Each subgroup in the analysis contained at least 392 samples.

The activity of all four enzymes generally remains stable for 12–24 h after birth before gradually declining through 96 h after birth.

### 4.3. Relationship Between Birthweight and Age at Collection

**Figure 7:**
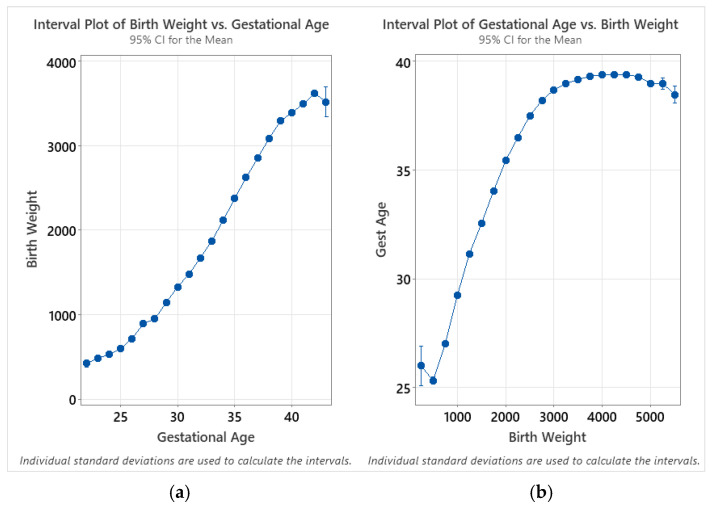
Interval plots of (**a**) birthweight as a function of gestational age; (**b**) gestational age as a function of birthweight. Individual group standard deviations used to calculate the intervals. Data are distributed by week of gestational age (22–43 weeks) and in 250 g intervals (250–5500 g).

Trends for mean enzyme activity are generally very consistent between birthweight and gestational age; this is expected as there is a close correlation between the two variables. However, while the mean activities are highest for the lowest-age-at-collection group, they are highest between 500 and 999 g. This can be potentially explained through analysis of interval plots relating the birthweight and gestational age for all subgroups, where the total number of samples is greater than 30 (gestational ages between 22 and 43 weeks—and birthweights between 250 and 5749 g; Figure 7). When calculating the confidence intervals for birthweight as a function of gestational age, the relative variability for each subgroup is small, with the exception of 43 weeks of gestational age, which showed a larger confidence interval due to the small sample size (*n* = 36). When the intervals are calculated for gestational age as a function of birthweight, however, the mean for the 250 g birthweight group is higher than for the 500 g group—and the large confidence interval for this group is representative of both a smaller sample size (*n* = 97) and a larger variance. This discontinuity explains the difference in the trends between charts at the low end of gestational age and birthweight.

While the overall trends are comparable between the two analyses, birthweight appears to provide a more reliable indicator in terms of smoothness of the trends as well as reduced variability within each subgroup. This is likely due to the unreliability of reported gestational age, as there is acknowledged unreliability of estimates of gestational age based on the last menstrual period (LMP). It is not specified in the Missouri dried blood spot specimen record whether the LMP or ultrasound are used for pregnancy dating when completing the sample collection form.

### 4.4. Activity for LBW and VLBW Samples After 14 and 28 Days

**Figure 8:**
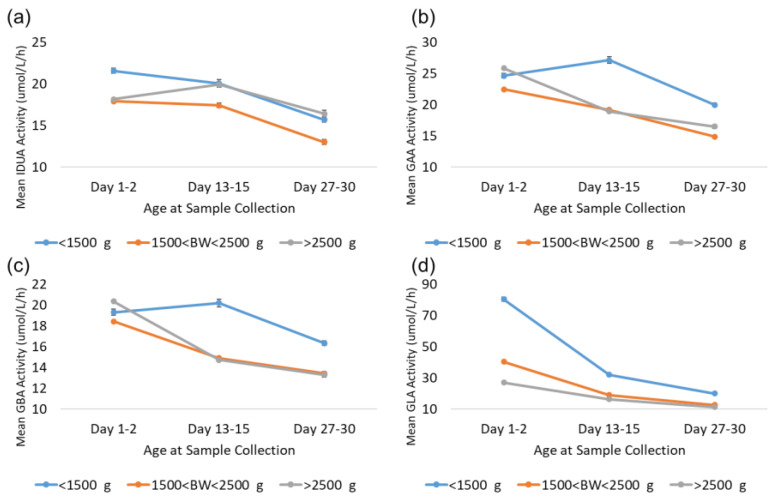
Enzyme activity by age at collection for VLBW, LBW, and normal-weight births for (**a**) IDUA, (**b**) GAA, (**c**) GBA, and (**d**) GLA. Datapoints represent the mean activity over a four-hour block of time. Error bars represent the 95% confidence interval for each subgroup.

As noted in the results, GLA enzyme activity is elevated in LBW and VLBW newborns relative to normal birthweight newborns (>2500 g). The Missouri code of state regulations (19 CSR 25–36.010) requires that in the case of premature infants who are less than 2000 g or less than 34 weeks of gestational age at birth, a repeat specimen should be collected between 7 and 14 days after birth and another repeat specimen should be collected at 28 days after birth [17,18]. Additional analyses were completed to investigate the back-transformed mean activity for LBW and VLBW newborns compared to normal-weight newborns over the first month to determine whether the mean activity for LBW and VLBW newborns reverts to the mean for normal-birthweight newborns at these age milestones. These analyses focused on three groups based on age at collection: 1–2 days, which represents typical collection; 13–15 days, which represents the second screen; and 27–30 days, which represents the third screen. These narrow ranges were selected to allow for some flexibility around sample collection date, while still reducing variability caused by using samples from a broad range of collection dates. Samples were additionally grouped by birthweight: VLBW (<1500 g), LBW (1501–2500 g), and normal (>2500 g). Given the large source dataset, each subgroup contained at least 1900 specimens. The results from these analyses for all four enzymes are displayed in Figure 8.

The trends observed for GAA and GBA were very similar; activity from VLBW, LBW, and normal-birthweight specimens were comparable from the first screen. LBW and normal-birthweight specimens’ mean activity decreased comparably at approximately 14 and 28 days, while VLBW specimen mean activity was 20–40% higher than that of normal-birthweight specimens at 14 and 28 days. For IDUA, VLBW specimens had approximately 20% higher mean activity than LBW and normal-birthweight specimens at 1–2 days. At day 14, mean IDUA activity for normal-birthweight specimens increased and was comparable to VLBW specimens at day 14 and 28, while the mean activity for LBW specimens was approximately 20% lower than that of either VLBW or normal-weight specimens at day 14 and 28.

For GLA, the mean for LBW specimens was approximately 50% higher than that for normal-birthweight newborns at day 1–2, but only 10–20% higher at day 14 and 28. VLBW specimens, however, remained significantly elevated compared to both LBW and normal-birthweight specimens. At 1–2 days, VLBW specimen mean activity was elevated by almost 200% relative to normal birthweight. At day 14, the VLBW mean was still almost 100% higher than the normal mean, and at day 28, the VLBW mean was still 75% higher than the normal mean. This suggests that VLBW newborns may still have significantly elevated GLA activity relative to the normal-birthweight population throughout the first month of life.

## 5. Conclusions

The Missouri newborn screening program operates the longest continuous screening program in the United States for MPS I, Pompe, Gaucher, and Fabry diseases. The screening program has been very successful. To date, more than 200 newborns have been positively identified with one of the four disorders, at a combined incidence rate of 1 in 2311. The clinical results of the screening program have been previously published [6,7,19,20]. When implementing screening for LSDs, it is important to consider the effect of demographic variables on lysosomal enzyme activity. The false positive rate associated with LSD screening is highly dependent on these demographic variables, as well as the rate of pseudodeficiency alleles, carrier status, and seasonal variability in enzyme activity. While laboratories commonly conduct pilot studies prior to beginning prospective screening, it is difficult to test a sufficient number of samples from each demographic subgroup to completely examine the correlation of these variables with statistical significance. Laboratories should consider utilizing several tools when implementing their first-tier cutoffs, including implementation of age-related cutoffs to reduce false positive rates. Additionally, as the lysosomal enzymes are subject to denaturation at elevated heat and humidity, cutoffs may be adjusted throughout the year, or floating cutoffs such as percentile of the daily mean could be implemented. Use of post-analytical tools such as Collaborative Laboratory Integrated Reports (CLIRs), which were developed to use covariate-adjusted percentiles based on age, birthweight, and gender, can further reduce the false positive rate by incorporating these variables into the evaluation of enzyme activity. Second tier biochemical or molecular screening can also be implemented to minimize false positive rates. As screening expands to include additional LSDs, laboratories should consider testing across these demographic variables to understand the trends for each lysosomal enzyme.

## Data Availability

The raw data supporting the conclusions of this article will be made available by the authors on request.
